# Bacterial Diversity and Antibiotic Resistance Patterns of Community-Acquired Urinary Tract Infections in Mega Size Clinical Samples of Egyptian Patients: A Cross-Sectional Study

**DOI:** 10.7759/cureus.51838

**Published:** 2024-01-08

**Authors:** Amr Tarek, Salah Abdalla, Nehal A Dokmak, Ali A Ahmed, Taghrid S El-Mahdy, Nesreen A Safwat

**Affiliations:** 1 Microbiology and Immunology, Modern University for Technology and Information (MTI), Cairo, EGY; 2 Microbiology and Immunology, Suez-Canal University, Ismailia, EGY; 3 Microbiology and Immunology, Helwan University, Cairo, EGY

**Keywords:** egypt, surveillance data, extended-spectrum beta-lactamases (esbl), multidrug resistance (mdr), antimicrobial susceptibilities, klebsiella species, escherichia coli, urinary tract infection (uti)

## Abstract

Background: Community-acquired urinary tract infection (UTI) is one of the most common infectious diseases nowadays. Alarming increased levels of antimicrobial resistance are developing globally which limit treatment options and may lead to life-threatening problems.

Aim: Our study aimed to collect surveillance data on non-hospitalized Egyptian UTI cases and to develop strategies against multidrug-resistant pathogens (MDR). According to our knowledge, this is the first study to screen this high number (15,252 urine samples) in a short period (three months), providing valuable data on resistance profiles in non-hospitalized Egyptian UTI patients.

Methods: A total of 15,252 urine samples were collected from different patients. Positive cultures were identified using a semi-quantitative method. Kirby-Bauer's disc diffusion method was used for antibiotic susceptibility testing, the double disc diffusion method was used for extended-spectrum beta-lactamases-producing strains, and the Chi-square test was used for statistical data processing.

Results: The results showed 61% positive cultures, females accounted for 67.5%. Infants and elderly patients showed the highest positive cultures (74.4% and 69.2%, respectively). Despite *Escherichia coli* being the most common uropathogen (47.19%), *Klebsiella *species(24.42%) were the most MDR and extended-spectrum β-lactamase (ESBL)-producing organisms. *E. coli* and *Klebsiella *spp. displayed increased resistance to cephalosporins (75% and 81%, respectively). In contrast, both organisms displayed high sensitivity to carbapenems. Unlike *Klebsiella *spp., *E. coli *was highly sensitive (92%) to first-line treatment (nitrofurantoin) for UTI. Moreover, trimethoprim/sulfamethoxazole showed higher sensitivity rates compared to other nations.

Conclusion: Despite *Escherichia coli* being the most often identified bacteria in our isolates *Klebsiella* spp. displayed higher resistance to the majority of tested antibiotics. Fortunately, trimethoprim/sulfamethoxazole significantly increased sensitivity, especially against* E. coli.* However, both species showed high rates of cephalosporin resistance. Moreover, It is important to promote Egypt's national action plan for antimicrobial resistance in collaboration with the World Health Organization, especially in the community to minimize the chance of bacterial resistance in the Egyptian community.

## Introduction

Urinary tract infection (UTI) is now one of the most common infectious illnesses in the world, with chronic and recurring infections posing significant challenges [1]. UTIs affect around 250 million individuals annually, accounting for roughly 40% of all infections globally [2]. Treatment of UTI is critical since its complications can greatly impact a patient's health and level of life and have a considerable effect on healthcare and economic costs [3]. The most common UTI complications, such as proteinuria, high blood pressure, and kidney damage, can all come from a lack of renal function [4]. Female contraception use, personal hygiene issues, white ethnicity, a past previous UTI, a lack of water intake, neurological bladder disease, having diabetes, genitourinary instruments, being born with genitourinary abnormalities, phimosis, incomplete/infrequent urination, and prolonged constipation are all risk factors for UTI [5]. Gram-negative pathogens such as* E. coli *and *Klebsiella *spp are responsible for most UTIs [6]. However, an alarming level of antimicrobial resistance develops in UTI pathogens because of the indiscriminate and widespread use of antibiotics. Appropriate treatment of UTIs has become challenging due to high resistance to commonly prescribed antibiotics [1]. In the African region, UTIs were reported as a highly incident risk infectious disease [7]. Therefore, this study aimed to screen urine samples taken from UTI Egyptian non-hospitalized patients for positive cultures and describe antibiotic resistance profiles of the most common isolates.

## Materials and methods

Samples collection

A total of 15,252 urine samples related to different patients from various regions and governorates in Egypt were collected from outpatient laboratories from March 2022 to May 2022. Midstream urine samples were collected from infected patients of different ages in both genders. Male patients were instructed to clean around the urethra meatus to minimize the risk of contamination. In the case of uncircumcised male patients, they were advised to retract the foreskin. Female patients were instructed to part the labia and clean from the front to back. Both male and female participants were instructed to expel 15-30 ml of urine into the toilet to eliminate any bacteria colonizing at the far end of the urethra. They were then advised to collect the midstream portion of the urine stream in a sterile container with a wide opening and dispose of the remaining urine in the toilet. For infants, the instructions were to thoroughly cleanse the skin in the genital region using soap and water or cleansing wipes. Then, first, let a small amount of urine flow into the toilet and then collect the midstream portion during urination. Any contact between the sample container and the skin was instructed to be avoided. The samples collected from various branches across Egypt of a mega medical laboratory were refrigerated at 4°C and then transported in an ice pack to the main core branch of the medical laboratory for analysis within 24 hours of collection.

Inclusion criteria

Non-hospitalized, urinary tract-infected Egyptian patients from the community with documented age, gender, and UTI symptoms such as dysuria, frequent urination, and whose urine was cloudy or bloody with a strong odor were included in the study. Confirmation of a urinary tract infection (UTI) was established through a positive culture, specifically when the colony count exceeded 10^5^ CFU/ml. Only samples collected following the Centers for Disease Control and Prevention (CDC) urine collection guidelines were included in the study.

Exclusion criteria

Non-Egyptian patients were not included in the study. Frozen urine samples or those provided in non-sterile containers are also excluded. Patients previously taken part in this study, currently using antibiotics at the time of the specimen collection and catheterized patients were ineligible to participate in this research.

Phenotypic identifications of microorganisms

The pathogenic bacterial strains were isolated from the positive collected urine samples and identified using the semi-quantitative method. Briefly, collected urine samples (1 μL - 10 μL) were used to inoculate MacConkey agar and nutrient agar plates supplemented with 5% sheep blood and incubated for 24 hours at 37°C. All microorganisms isolated from positive cultures (≥10^5^ CFU/mL) were identified using Gram staining, cultural characteristics, and different biochemical tests [8].

Antimicrobial susceptibility testing

Antibiotic susceptibility testing of collected fresh cultures of *E. coli* and *Klebsiella* spp was performed using Kirby-Bauer’s disc diffusion method by which, the tested organisms were prepared into McFarland tube no. 0.5; inoculum density 1.5 ×10^8^ organisms/mL. Then, the culture was spread evenly over the Muller Hinton agar surface. After covering the test organism culture with the antibiotic discs, the plates were incubated for 24 hours at 37˚C. The zone of inhibition's diameter was measured after incubation. The results were classified as "Resistant," or "Sensitive" to antibiotics, according to the Clinical and Laboratory Standards Institute (CLSI, 2021). Prescribed antibiotic discs related to different classes were assessed for sensitivity tests (Oxoid, UK), namely imipenem (IPM; 10 µg), meropenem (MEM; 10 µg), ertapenem (ETP; 10 μg), amikacin (Ak; 30 µg), gentamicin (CN; 10 µg), piperacillin/tazobactam (TZP; 110 μg), nitrofurantoin (F; 300μg), sulfamethoxazole (SXT; 1.25/23,75 μg), levofloxacin (LEV; 5 μg), cefepime (FEP; 30 μg), cefotaxime (CTX; 30 μg), cefuroxime (CXM; 30 µg), Cefazolin (CZ; 30 µg) ceftazidime (CAZ; 30 µg), ampicillin/sulbactam (SAM; 10/10 µg), ampicillin (AMP; 10µg) The *E. coli* and *Klebsiella* spp bacterial isolates were classified as multidrug-resistant (MDR) when they demonstrated resistance to one agent in three or more antimicrobial classes [9].

Detection of extended-spectrum beta-lactamases (ESBL) producing strains (double disc diffusion testing)

The collected isolates of *Klebsiella *spp and *E. coli* were tested for beta-lactamase production according to CLSI guidelines. Discs holding 30 µg of ceftazidime or ceftriaxone were assessed against the tested isolates alongside amoxicillin-clavulanic acid discs (10 µg). Following a 24-hour incubation at 37˚ C, an increase in the inhibition zone was related to an ESBL-producing microorganism.

Statistical analysis

Data analysis was performed by SPSS version 23 for Windows (IBM Corp., Armonk, NY). The Chi-square test was used to determine the association between positive culture with age groups, and gender. Moreover, the Chi-square test is used to determine whether there is a statistically significant association between *E. coli* and *Klebsiella *spp in producing ESBL and MDR character. The antimicrobial resistance results of *E. coli* and *Klebsiella *spp in different demographic variables were examined by Chi-square test and cells with a count less than five values were tested with Fisher’s exact test. *P* values were two-sided, with *p *< 0.05 regarded as statistically significant.

Ethical statement

Our study was approved by the Institutional Review Board (IRB) (No.202203m1) at the Suez Canal University and consent was obtained by all participants in this study.

## Results

Positive growth culture distribution

**Figure 1:**
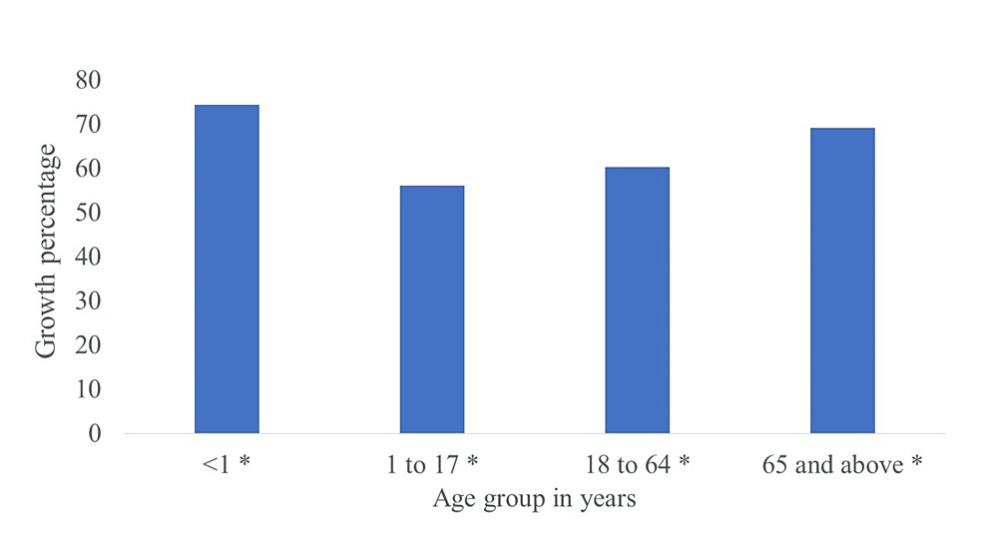
Percentage of positive culture in each age group *Significant associations exist between different age groups and positive culture at *p *< 0.05.

Of 15,252 urine samples collected from patients, 9317 showed a positive bacterial culture (61%). A higher percentage of positive culture was reported in women 67.5% (7691/11389) than that collected from men's urine samples 42.1% (1626/3863) and these results were statistically significant at *p *< 0.05 (Table 1). Age groups under one year and older than 64 years displayed a high percentage of a positive culture with 74.4% and 69.2%, respectively. In contrast, the childhood age group (1-17 years) showed the lowest rate of positive cultures among other groups (Figure 1).

**Table 1:** Number (%) of positive cultures in each age group

Age group (years)	Number of positive cultures		
	Male N=3863	Female N=11389	Total number
<1	34(55.7%)	27(44.2%)	61
1-17	180(14.9%)	1022(85%)	1202
18-64	855(13.2%)	5608(86.7%)	6463
65 and above	557(35%)	1034(64.9%)	1591
Total number	1626(42.1%)	7691(67.5%)	9317

Distribution of microorganisms in the collected positive cultures

**Figure 2:**
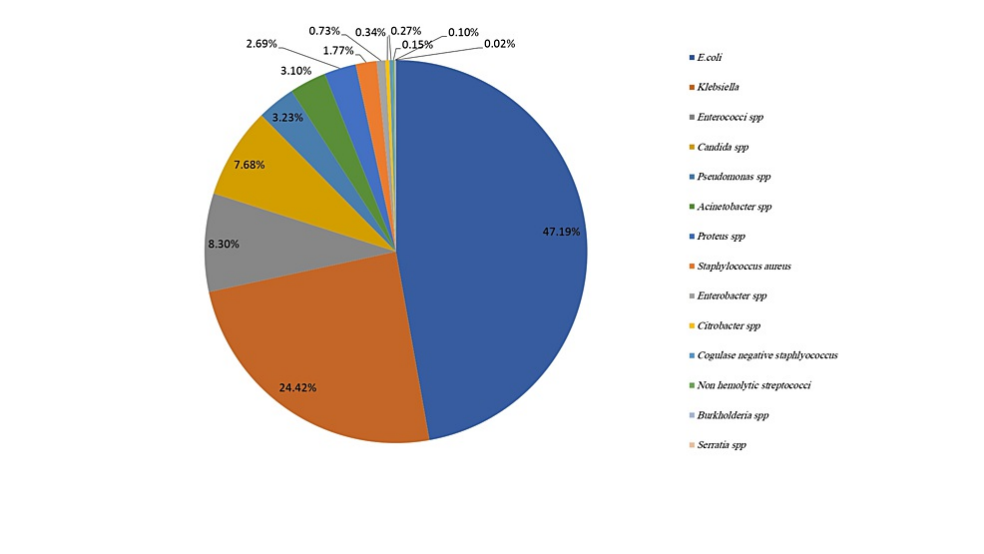
Distribution of microorganisms in positive culture urine samples

The results revealed that Gram-negative bacteria were the most common cause of urinary tract infections (UTIs) in the collected positive samples (81.1%). The Enterobacteriaceae family accounts for 92.2% of Gram-negative bacteria with a high prevalence of *E. coli* and *Klebsiella* spp. Other microorganisms, such as *Enterobacter* spp and *Citrobacter* spp were recovered in lower percentages (Figure 2 and Table 2). The growth of* E. coli* and *Klebsiella* spp showed different demographic variables where much higher incidences of females in both pathogens (86.4%, and 84.1%, respectively) than in males. Moreover, the prevalence of *E. coli *in* *UTI samples was higher than that of *Klebsiella* spp in all female age groups.

**Table 2:** Number and percentage of microorganisms out of 9317 positive urine cultures

Gram negative bacilli (N=7624) (81.8%)				Gram positive cocci (N=977) (10.4%)		Fungus (N=716) (7.6%)	
Enterobacteriaceae (7025) (75%)		Non Enterobacteriaceae (599) (6.4%)		*Enterococci* spp	773 (8.2%)	*Candida *spp	716 (7.6%)
E. coli	4397 (47%)	*Pseudomonas* spp	301 (3.2%)	*Staphylococcus aureus *	165 (1.77%)		
*Klebsiella* spp	2275 (24.5%)	*Acinetobacter *spp	289 (3.1%)	Coagulase-negative* Staphylococcus*	25 (0.26%)		
*Proteus* spp	251 (2.6%)	*Burkholderia *spp	9 (0.09%)	Non hemolytic* Streptococci *	14 (0.15%)		
*Enterobacter* spp	68 (0.7%)						
*Citrobacter* spp	32 (0.3%)						
*Serratia *spp	2 (0.02%)						

Females were highly susceptible to UTI by *Klebsiella* spp at the age of 18 to 64 years compared to other age groups. While females in age groups 1 to 17 years and 18 to 64 years were the most infected with E. coli. Infant males less than one year old were the most infected by both UTI pathogens among males in other age groups, as recorded in Table 3.

**Table 3:** The positive growth cultures of E. coli and Klebsiella spp in different demographic variables

Age (Years)	E. coli			Age	*Klebsiella *spp		
	Male (%)	Female (%)	Total number (%)		Male (%)	Female (%)	Total number (%)
<1	9 (41%)	13 (59%)	22 (36%)	<1	13 (56.5%)	10 (43.5%)	23 (37.7%)
1-17	69 (10%)	620 (90%)	689 (57.3%)	1-17	32 (15.3%)	177 (84.7%)	209 (17.3%)
18-64	300 (10%)	2620 (90%)	2920 (45.1%)	18-64	170 (10.3%)	1477 (89.7%)	1647(25.4%)
>64	218 (28.5%)	548 (71.5%)	766 (48.1%)	>64	146 (36.9%)	250 (63.1%)	396 (24.9%)
Total number (%)	596(13.5%)	3801(86.4%)	4379	Total number. (%)	361(15.8%)	1914(84.1%)	2275

Antimicrobial susceptibility test and ESBL production of *E. coli* and *Klebsiella* spp.

**Figure 3:**
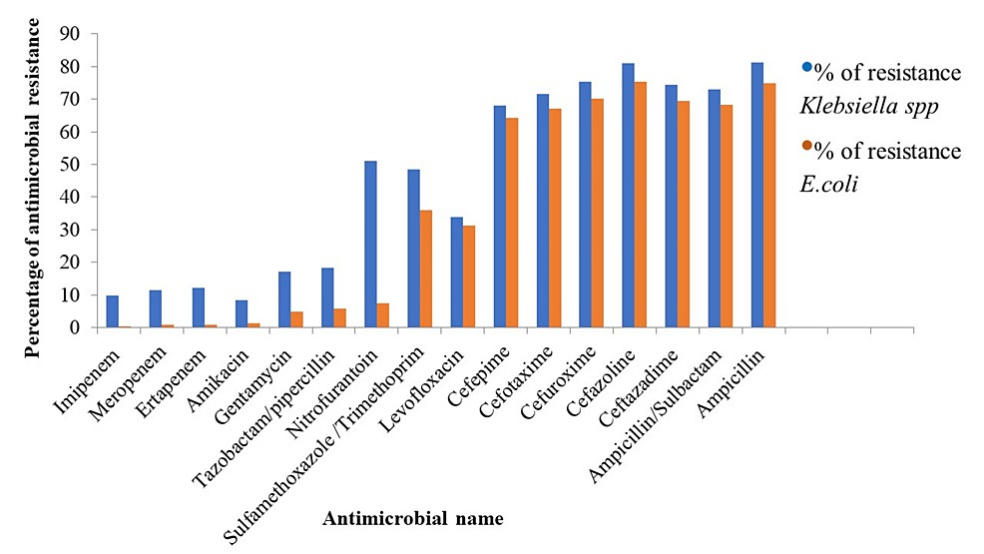
Percentage of resistance of isolated E. coli and Klebsiella spp to different antimicrobial classes.

The results of the antimicrobial susceptibility test showed a higher prevalence of MDR isolates in *Klebsiella *spp than in *E. coli* (43.4% Vs. 30.3%, respectively) and these results were statistically significant at *p *< 0.05. The tested isolates of* E. coli *and *Klebsiella *spp were highly resistant to cefuroxime (70.2%, 75.3%), cefazoline (75.3%, 81%), ceftazidime (69.4%, 74.4%), and ampicillin (74.7%, 81.3%); respectively. On the other hand, *E. coli* and *Klebsiella* spp showed less resistance rates against imipenem (0.38%, 9.7%), meropenem (0.7%, 11.4%), ertapenem (0.7%, 12.1%), amikacin (1.2%, 8.3%); respectively. Moreover, nitrofurantoin as a first-line treatment of UTIs remains effective for *E. coli* with a susceptibility level of 92%, whereas its sensitivity level was only 49% in *Klebsiella* spp (Figure 3). *E. coli* was sensitive to sulfamethoxazole/trimethoprim with a percentage of 64%. The male infants infected by *E. coli* or *Klebsiella* spp were the most resistant age group to carbapenem, cephalosporins, and nitrofurantoin, while female infants infected by *E. coli* were most resistant to amikacin, gentamycin, and tazobactam/piperacillin antibiotics. Patients of both genders above 64 years old were the most resistant to levofloxacin and ampicillin (Table 4). The percentage of ESBL production in *E. coli *and *Klebsiella* spp were as high as 59% and 63%, respectively with a statistically significant association at a *p-*value equal to 0.001.

**Table 4:** The antimicrobial resistance number (%) of E. coli and Klebsiella spp isolates in different demographic variables. *Significant difference between *E. coli* and *Klebsiella* spp in antibiotic resistance result (*p* <0.05)

Antibiotic/Age group (year)	E. coli				*Klebsiella* spp			
	<1	1-18	18-64	>64	<1	1-18	18-64	>64
Imipenem								
Male N (%)	1/9 (11%)	1/68 (1.4%)	0/300* (0%)	3/218* (1.3%)	5/13 (38.4%)	4/32* (12.5%)	37/170* (22%)	40/146* (27%)
Female N (%)	0/13 (0%)	1/620* (0.16%)	4/2620* (0.15%)	7/548* (1.2%)	0/10 (0%)	15/177* (8.4%)	76/1477* (5.14%)	45/250* (18%)
Meropenem								
Male N (%)	1/9 (11%)	1/69* (1.4%)	2/300* (0.6%)	6/218* (2.7%)	6/13 (46.1%)	4/32* (12.5%)	40/170* (23.5%)	43/146* (29.4%)
Female N (%)	0/13 (0%)	5/620* (0.8%)	9/2620* (0.3%)	8/548* (1.4%)	0/10 (0%)	21/177* (11.8%)	93/1477* (6.2%)	54/250* (21.6%)
Ertapenem								
Male N (%)	1/9 (11.1%)	1/69* (1.4%)	1/300* (0.3%)	4/218* (1.8%)	6/13 (46.1%)	4/32* (12.5%)	43/170* (25.2%)	46/146* (31.5%)
Female N (%)	1/13 (7.6%)	4/620* (0.6%)	14/2620* (0.5%)	6/548* (1%)	1/10 (10%)	19/177* (10.7%)	102/1477* (6.9%)	56/250* (22.4%)
Amikacin								
Male N (%)	0/9 (0%)	0/69* (0%)	5/300* (1.6%)	10/218* (4.5%)	4/13 (30.7%)	3/32* (9.3%)	33/170* (19.4%)	39/146* (26.7%)
Female N (%)	3/13 (23%)	4/620* (0.6%)	27/2620* (1.03%)	7/548* (1.2%)	0/10 (0%)	8/177* (4.5%)	62/1477* (4.1%)	41/250* (16.4%)
Gentamycin								
Male N (%)	1/9* (11.1%)	1/69* (1.4%)	22/300* (7.3%)	28/218* (12.8%)	8/13* (61.5%)	7/32* (21.8%)	48/170* (28.2%)	57/146* (39%)
Female N (%)	3/13 (23%)	29/620* (4.6%)	102/2620* (3.8%)	28/548* (5.1%)	2/10 (20%)	27/177* (15.2%)	169/1477* (11.4%)	72/250* (28.8%)
Tazobactam/pipercillin								
Male N (%)	1/9* (11.1%)	6/69 (8.8%)	17/300* (5.6%)	17/218* (7.7%)	8/13* (61.5%)	5/32 (15.6%)	60/170* (35.2%)	66/146* (45.2%)
Female N (%)	3/13 (23%)	38/620* (6.1%)	128/2620* (4.8%)	42/548* (7.6%)	1/10 (10%)	31/177* (17.5%)	173/1477* (11.7%)	73/250* (29.2%)
Nitrofurantoin								
Male N (%)	3/9 (33.3%)	5/69* (7.35%)	23/300* (7.6%)	20/218* (9.1%)	9/13 (69.2%)	12/32* (37.5%)	109/170* (64.1%)	94/146* (64.3%)
Female N (%)	1/13* (7.6%)	41/620* (6.6%)	184/2620* (7%)	50/548* (9.1%)	9/10* (90%)	82/177* (46.3%)	709/1477* (48%)	139/250* (55.6%)
Sulphamethoxazole/trimethoprim								
Male N (%)	3/9 (33.3%)	23/69 (33.8%)	119/300* (39.6%)	96/218* (44%)	9/13 (69.2%)	14/32 (43.7%)	101/170* (59.4%)	94/146* (64.3%)
Female N (%)	5/13 (38.4%)	235/620* (37.9%)	868/2620* (33.1%)	233/548* (42.5%)	6/10 (60%)	87/177* (49.1%)	645/1477* (43.6%)	147/250* (58.8%)
Levofloxacin								
Male N (%)	2/9* (22.2%)	10/69* (14.7%)	141/300* (47%)	141/218 (64.6%)	9/13* (69.2%)	11/32* (34.3%)	103/170* (60.5%)	98/146 (67.1%)
Female N (%)	4/13 (30.7%)	71/620* (11.4%)	747/2620* (28.5%)	261/548* (47.6%)	3/10 (30%)	53/177* (29.9%)	347/1477* (23.4%)	143/250* (57.2%)
Cefepime								
Male N (%)	7/9 (77.7%)	37/69 (54.4%)	207/300* (69%)	162/218* (74.3%)	12/13 (92.3%)	23/32 (71.8%)	132/170* (77.6%)	122/146* (83.5%)
Female N (%)	10/13 (76.9%)	402/620 (64.8%)	1620/2620 (61.8%)	377/548 (68.7%)	8/10 (80%)	121/177 (68.3%)	946/1477 (64%)	186/250 (74.4%)
Cefotaxime								
Male N (%)	8/9 (88.8%)	39/69 (57.3%)	211/300* (70.3%)	167/218 (76.6%)	12/13 (92.3%)	23/32 (71.8%)	141/170* (82.9%)	123/146 (84.2%)
Female N (%)	10/13 (76.9%)	423/620 (68.2%)	1699/2620 (64.84%)	390/548 (71.16%)	8/10 (80%)	131/177 (74%)	998/1477 (67.5%)	190/250 (76%)
Cefuroxime								
Male N (%)	8/9 (88.8%)	50/69 (73.5%)	215/300* (71.6%)	173/218 (79.3%)	12/13 (92.3%)	27/32 (84.3%)	145/170* (85.2%)	127/146 (86.9%)
Female N (%)	10/13 (76.9%)	473/620* (76.2%)	1760/2620* (67.1%)	398/548 (72.6%)	10/10 (100%)	148/177* (83.6%)	1050/1477* (71%)	196/250 (78.4%)
Cefazolin								
Male N (%)	9/9 (100%)	60/69 (88.2%)	226/300* (75.3%)	179/218* (82.1%)	12/13 (92.3%)	30/32 (93.75%)	152/170* (89.4%)	132/146* (90.4%)
Female N (%)	10/13 (76.9%)	528/620* (85.1%)	1884/2620* (71.9%)	415/548* (75.7%)	10/10 (100%)	164/177* (92.6%)	1135/1477* (76.8%)	210/250* (84%)
Ceftazidime								
Male N (%)	8/9 (88.8%)	45/69 (66.1%)	218/300* (72.6%)	170/218* (77.9%)	12/13 (92.3%)	26/32 (81.2%)	143/170* (84.1%)	129/146* (88.3%)
Female N (%)	10/13 (76.9%)	449/620* (72.4%)	1757/2620 (67%)	397/548 (72.4%)	10/10 (100%)	144/177* (81.3%)	1033/1477 (69.9%)	196/250 (78.4%)
Ampicillin/Sulbactam								
Male N (%)	7/9 (77.7%)	42/69 (61.7%)	208/300* (69.3%)	169/218 (77.5%)	11/13 (84.6%)	23/32 (71.8%)	143/170* (84.1%)	121/146 (82.8%)
Female N (%)	10/13 (76.9%)	416/620 (67%)	1748/2620 (66.7%)	400/548* (72.9%)	7/10 (70%)	129/177 (72.8%)	1021/1477 (69.1%)	207/250* (82.8%)
Ampicillin								
Male N (%)	7/9 (77.7%)	45/69* (66.1%)	229/300* (76.3%)	180/218 (82.5%)	12/13 (92.3%)	28/32* (87.5%)	150/170* (88.2%)	131/146 (89.7%)
Female N (%)	10/13 (76.9%)	468/620* (75.4%)	1926/2620* (73.5%)	423/548* (77.1%)	9/10 (90%)	151/177* (85.3%)	1149/1477* (77.7%)	220/250* (88%)

## Discussion

UTIs are serious public health problem that occurs in both males and females in all age stages. Globally, empiric therapeutic options for UTIs are limited by the emergence of multi-drug resistant uro-pathogens which may lead to treatment failures [10]. The results showed a high prevalence of *E. coli* and *Klebsiella* spp in the collected positive cultures isolated from UTI patients (47.19%, and 24.42%, respectively) followed by* Enterococcus* spp and *Candida* spp (approximately 8% each) as reported by other studies in different countries including Egypt [11] and Iran [12] with the percentage of *E. coli *(42.25% out of 232 isolates in Egypt, 58.82% out of 64 isolates in Iran) and *Klebsiella* spp (19.4% out of 232 isolates in Egypt, 19.12% out of 64 isolates in Iran). 

The incidence of infection in our study was higher in females than in males, where the percentage of growth of* E. coli *and* Klebsiella* spp in females’ urine samples were 86.4% and 84.1%, respectively compared to 13.5% and 15.8%, respectively in males. These results may be attributed to several clinical factors including anatomical differences between both males and females, hormonal effects, behavior patterns, and the female urethra's physiological and anatomical properties [13]. In agreement with our results, females were more susceptible to UTI than males in Egypt and Portugal [11,14]. Males were less susceptible to UTIs because the male urethra is longer, creating an obstacle that keeps bacteria out of the urinary bladder [15].

In our study, the urine samples of people above 64 years old and infants (less than one year) had the highest percentage of a positive culture (69.2% and 74.4%, respectively). Infants accounted for 57.4% of the positive cultures in another study [16]. It was reported that old patients were the most affected by UTIs, with a frequency of 56.9% [14]. Moreover, Xie et al. reported that the Chinese senior population over 60 years had the highest percentage of UTIs [17]. The prevalence of UTIs may be increased in old age due to decreased immunity, reduced estrogen levels, and decreased water intake in old age [13,18]. Infant UTI may be attributed to congenital blockage, Phimosis, ureterovesical valve failure, undeveloped host defenses, and exposure to microorganisms that can enter the urinary system by fecal soiling [19]. It is worth mentioning that in our study, the male infants showed a higher total number of positive cultures (56%) than female infants (44%). The difference might be attributed to young males' lack of circumcision at this age [20]. Interestingly, our statistical results showed that Egyptian infants (less than one year) were equally susceptible to *E. coli* and *Klebsiella* spp (36% and 38%, respectively). In contrast, there was a significant difference between the percentage of *E. coli* (68.6% out of 242 samples) and* Klebsiella* spp (10.3% out of 242 samples) in urine samples of infected Bahrani infants [5]. This difference may be due to different research designs, environmental conditions, or patient selection criteria.

The antibiotic sensitivity test was demonstrated to investigate the possible therapeutic options of UTI patients in different test groups. The results showed that 30.3% of the *E. coli* isolates and 43.4% of *Klebsiella* spp could be described as multi-drug-resistant isolates. This agreed with a study in Ethiopia, the MDR pattern was higher in *Klebsiella* spp (93.3%) than in *E. coli* (80.4%) [21]. In addition, *Klebsiella* spp was more common as an ESBL-producing UTI pathogen than* E. coli* (63.4% and 59.1%, respectively) in our study. This finding agreed with other studies in different countries such as Ethiopia [21], and Saudi Arabia [22] by which *Klebsiella* spp was the most ESBL producer gram-negative microorganism with percentages of *Klebsiella* spp (86.7%) vs* E. coli* (52.2%), and *Klebsiella* spp (48.7%) vs *E. coli* (41.9%), respectively. There are limited oral options for treating ESBL-producing bacteria associated with UTI. Therefore, in this study, a wide margin of samples of* E. coli* and *Klebsiella* spp (4379 and 2275, respectively) was included for the first time in Egypt which led to realistic outputs. In this study, high resistance rates were recorded against the typical antibiotic used to treat UTIs in the Egyptian community. A markedly increased resistance of* E. coli* and *Klebsiella* spp to different cephalosporin generations (75% and 69%, respectively) and ampicillin (74% and 81%, respectively) were obtained. Our results were alarming the high resistance pattern of both *E. coli* and *Klebsiella* spp towards the commonly used generations of cephalosporins, which were higher than that in other countries, as the average resistant pattern was 60% in another study conducted in Egypt [11], but only 54% in Nepal [23],40% in South Africa [10], and 24% in Taiwan [24]. Moreover, the Egypt national action plan for antimicrobial resistance https://www.who.int/publications/m/item/egypt-national-action-plan-for-antimicrobial-resistance reported that the average resistant pattern in hospitalized patients was about 86% for both *E. coli *and *Klebsiella* spp. These findings may be due to high prescriptions of cephalosporins in Egypt, either by physician or pharmacist recommendations [25]. However, in *Klebsiella *spp and *E. coli*, the addition of beta-lactamase inhibitors only resulted in an 8% and 6% increase in ampicillin sensitivity, respectively. In comparison to sulbactam/ampicillin, tazobactam/piperacillin, not available in Egyptian community pharmacies showed 94% sensitivity in *E. coli *and 82% sensitivity in *Klebsiella *spp [25]. In addition, we observed a high resistance pattern of *Klebsiella* spp of 51% to nitrofurantoin, a first-line treatment for UTIs. This may limit its use in the treatment of UTI for Egyptian patients. The male infants infected by *E. coli *or *Klebsiella* spp were the most resistant age group to carbapenem, cephalosporins, and nitrofurantoin, while female infants infected by *E. coli *were most resistant to amikacin, gentamycin, and tazobactam/piperacillin antibiotics. These results may be due to vertical transmission of antibiotic resistance from mothers to their offspring and consumption of antibiotics in early life [26]. In the current study, surprisingly there was a higher sensitivity to trimethoprim/sulfamethoxazole than in other countries, especially in *E. coli *(64%), this is higher than in a study from Taiwan, which had a 51% sensitivity pattern [24], and Turkey's 42% [27]. This may be explained by the low prescription frequency of trimethoprim/sulfamethoxazole in Egypt [25]. Therefore, we emphasized the role of trimethoprim/sulfamethoxazole in empirical antibiotics because of the recent decrease in the resistance rates in Egyptian governorates as the sensitivity pattern in a study conducted in 2009 in Egypt was 42 %[28]. However, it may not be possible to reuse the drug worldwide within the next several years, and close observation of surveillance data will be required.

Both isolates have a high sensitivity to carbapenems, with an average sensitivity pattern of 99.4% for *E. coli* and 89% for *Klebsiella* spp. These results are closely comparable to those obtained in Egypt and other nations, where the average sensitivity was 91% for *Klebsiella* spp and 98.5% for *E. coli* in Egypt [11], 84% for *Klebsiella* spp and 98% for *E. coli* in Turkey [27], and 92% for *Klebsiella* spp and 94% for *E. coli* in Taiwan [24]. Amikacin in our study is thought to be the most effective aminoglycoside, showing a sensitivity pattern of 91.7% in *Klebsiella* spp and 98.7% in *E. coli*, which is consistent with the results in Taiwan [24], where amikacin showed a sensitivity of 96.9% in *Klebsiella *spp and 98.8% in *E. coli*. Patients of both genders above 64 years old were the most resistant to levofloxacin and ampicillin and this may be because older individuals have a significant impact on the spread of multidrug-resistant organisms [29].

Limitations

This study exclusively focused on the culturable bacteria and yeast. The study primarily examined resistance patterns for solely the two most frequent uropathogenic bacteria,* Escherichia coli* and *Klebsiella *species, and the effect of gender and age groups on the prevalence of resistance. However, other factors contribute to the development of urinary tract infections (UTIs) as well as the prevalence of resistance, including the host's immune response, urinary tract abnormalities, virulence characteristics of microorganisms, and factors that disturb the equilibrium of the normal flora. It is important to consider these unidentified variables when interpreting the observed relationships between gender, age groups, and resistance patterns. Therefore, future research must be carried out to address these limitations.

## Conclusions

In this study, *Klebsiella *spp were more resistant to most of the tested antibiotics than* E. coli,* although *E. coli* is the most identified bacteria in our isolates (47%). Luckily, trimethoprim/sulfamethoxazole showed enhanced sensitivity, particularly by *E. coli *(64%). In contrast, high resistance rates were seen to cephalosporins by both organisms (75% and above). While the resistance level to nitrofurantoin (first line of UTI treatment) was 51% in *Klebsiella* spp, the drug remains effective for *E. coli *UTIs in males and females (92% sensitivity). Therefore, it is important to promote Egypt's national action plan for antimicrobial resistance in collaboration with WHO (https://www.who.int/publications/m/item/egypt-national-action-plan-for-antimicrobial-resistance) among various medical professionals in hospitals and the community to minimize the chance of bacterial resistance development especially among infants and elderly patients in the Egyptian community.
